# A Case-Control Study of the Genetic Variability in Reactive Oxygen Species—Metabolizing Enzymes in Melanoma Risk

**DOI:** 10.3390/ijms19010242

**Published:** 2018-01-14

**Authors:** Tze-An Yuan, Vandy Yourk, Ali Farhat, Argyrios Ziogas, Frank L. Meyskens, Hoda Anton-Culver, Feng Liu-Smith

**Affiliations:** 1 Program in Public Health, University of California Irvine, Irvine, CA 92697, USA; tzeany@uci.edu (T.-A.Y.); flmeyske@uci.edu (F.L.M.); 2 Department of Neurobiology and Behavior, School of Biological Sciences, University of California Irvine, Irvine, CA 92697, USA; vandyyourk@gmail.com; 3 Department of Biomedical Engineering, The Henry Samueli School of Engineering, University of California Irvine, Irvine, CA 92697, USA; farhatam@uci.edu; 4 Department of Epidemiology, School of Medicine, University of California, Irvine, CA 92697, USA; aziogas@uci.edu (A.Z.); hantoncu@uci.edu (H.A.-C.); 5 Chao Family Comprehensive Cancer Center, Irvine, CA 92697, USA

**Keywords:** melanoma, reactive oxygen species, ROS, NADPH oxidase, single nucleotide polymorphisms, SNP, superoxide dismutase, SOD2, SOD3, RAC1

## Abstract

Recent studies have shown that ultraviolet (UV)-induced chemiexcitation of melanin fragments leads to DNA damage; and chemiexcitation of melanin fragments requires reactive oxygen species (ROS), as ROS excite an electron in the melanin fragments. In addition, ROS also cause DNA damages on their own. We hypothesized that ROS producing and metabolizing enzymes were major contributors in UV-driven melanomas. In this case-control study of 349 participants, we genotyped 23 prioritized single nucleotide polymorphisms (SNPs) in nicotinamide adenine dinucleotide phosphate (NADPH) oxidases 1 and 4 (*NOX1* and *NOX4*, respectively), *CYBA*, *RAC1*, superoxide dismutases (*SOD1*, *SOD2*, and *SOD3*) and catalase (*CAT*), and analyzed their associated melanoma risk. Five SNPs, namely rs1049255 (*CYBA*), rs4673 (*CYBA*), rs10951982 (*RAC1*), rs8031 (*SOD2*), and rs2536512 (*SOD3*), exhibited significant genotypic frequency differences between melanoma cases and healthy controls. In simple logistic regression, *RAC1* rs10951982 (odds ratio (OR) 8.98, 95% confidence interval (CI): 5.08 to 16.44; *p* < 0.001) reached universal significance (*p* = 0.002) and the minor alleles were associated with increased risk of melanoma. In contrast, minor alleles in *SOD2* rs8031 (OR 0.16, 95% CI: 0.06 to 0.39; *p* < 0.001) and *SOD3* rs2536512 (OR 0.08, 95% CI: 0.01 to 0.31; *p* = 0.001) were associated with reduced risk of melanoma. In multivariate logistic regression, *RAC1* rs10951982 (OR 6.15, 95% CI: 2.98 to 13.41; *p* < 0.001) remained significantly associated with increased risk of melanoma. Our results highlighted the importance of *RAC1*, *SOD2*, and *SOD3* variants in the risk of melanoma.

## 1. Introduction

Ultraviolet (UV) rays are capable of inducing melanin production in melanocytes and promoting melanin transportation to the outermost layer of the skin—the keratinocytes. These melanins form a cap over the nucleus of both cell types and protect DNA from direct energy destruction [1,2]. On the other hand, UV rays are also able to initiate nicotinamide adenine dinucleotide phosphate (NADPH) oxidase (NOX) dominated reactive oxygen species (ROS) production and chemiexcitation of melanin fragments that affect DNA stability in melanocytes [3,4,5]. The oncogenic characteristics of UV-induced ROS signaling have not yet been fully elucidated, particularly in the transformation of melanocytes to melanomas. 

**Figure 1:**
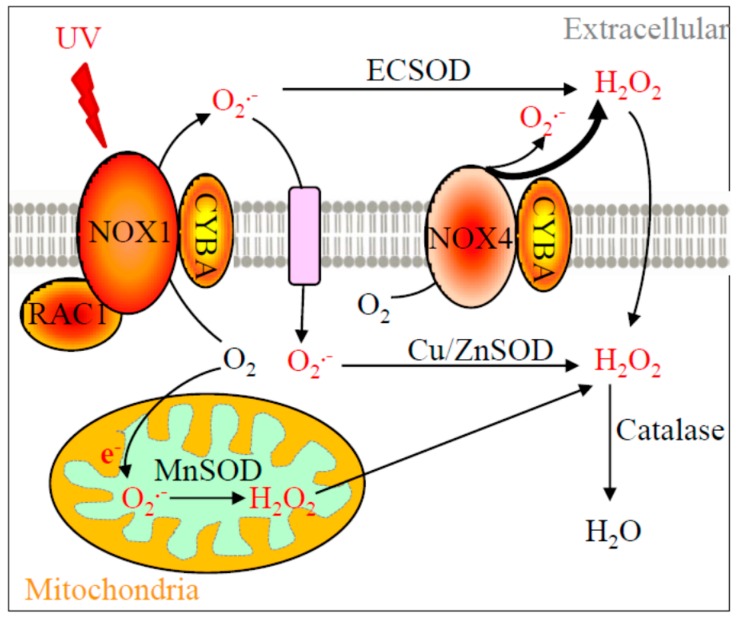
Diagram of the relevant reactive oxygen species (ROS) production pathway. NOX1, NOX4, CYBA, RAC1, SOD enzymes, catalase, their subcellular locations, and their functions in ROS production and metabolism are depicted in this diagram. NOX1 enzyme complex utilizes CYBA as one of its subunits and is activated by RAC1-GTPase to produce superoxide. On the other hand, NOX4 only couples with CYBA to generate hydrogen peroxide and superoxide. Of particular note, only plasma membrane NOX4 is shown in this diagram but mitochondrial or nuclear NOX4 has also been reported [16]. NOX1 is activated by UV to enhance its superoxide production, which requires the GTPase activity of RAC1. Superoxide is further metabolized into hydrogen peroxide at various subcellular locations by different SOD isozymes. Hydrogen peroxide is then converted into water molecules by catalase. Other additional redox enzymes (e.g., glutathione peroxidases, which also convert hydrogen peroxide into water) are not the focus in this study and therefore not included. Black arrows indicate the cellular movement of oxygen, ROS, and enzymatic metabolisms. A bold arrow represents a greater relative amount of ROS produced.

Recent understanding of melanoma photobiology has implied the etiological role of NOX enzymes, particularly NOX1 and NOX4 [6,7,8]. NOX enzymes produce superoxide and/or hydrogen peroxide when coupled with CYBA (p22phox) membrane protein [9]. RAC1, a newly defined melanoma oncogene [10], is shown to enhance NOX1 activity [11]. The downstream ROS metabolizing enzymes, e.g., copper-zinc superoxide dismutase (Cu-ZnSOD, *SOD1*), manganese superoxide dismutase (MnSOD, *SOD2*), and extracellular superoxide dismutase (ECSOD, *SOD3*), convert superoxide to hydrogen peroxide. Catalase then transforms hydrogen peroxide to water molecules (Figure 1). The cellular locations of NOX1, RAC1, NOX4, CYBA, and SOD enzymes, and their functions in ROS production and metabolism are illustrated in Figure 1. Little is known about the comprehensive role of this entire pathway in melanoma formation. However, risk associated with these genes has been reported in various health conditions. For example, V16A variant in *SOD2* (rs4880) showed an impaired mitochondrial importing function and was associated with prostate cancer risk [12]. The rs7277748 and rs4998557 variants in *SOD1* were found to be associated with amyotrophic lateral sclerosis [13]. Variants rs2536512 and rs699473 in *SOD3* were linked to cerebral infarction [14] and brain tumor [15]. 

Although the causal network of melanoma has not yet been fully elucidated [17], UV exposure is the most tangible environmental risk factor that can be readily modified by behavioral precautions [18]. Therefore, the purpose of this study was to explore the relationship between the hypothesized photobiological pathway and risk of melanoma. Specifically, our aim was to use the candidate gene approach to discover the association of variations in the genetic profile of the redox enzymes with melanoma (Figure 1). Building upon this rationale, functional genetic variants, namely single nucleotide polymorphisms (SNPs), were identified in this study with a priori chance of being associated with the risk of melanoma based on the following criteria: (1) not a well-known somatic mutation found in tumors with an established causality; (2) presented strong associations with many other health conditions in humans; and (3) with a potential to alter normal protein function based on the nucleotide substitution. For instance, variant rs8031 in *SOD2* was found to be associated with kidney complications in subjects with Type 1 diabetes [19]. Variant rs10951982 in *RAC1* has been implied in the increased risk of hypertension [20]. Even though rs10951982 in *RAC1* has not yet been reported in ROS-related malignancies, somatic mutations of *RAC1* (e.g., *RAC1*^P29S^) were found in 9.2% of sun-exposed melanoma tumors [21,22].

With this genetic profiling information in hand, we hope to lay a foundation to identify those individuals predisposed to UV exposure and risk of melanoma. This in turn will contribute to a better primary prevention strategy, such as earlier-life behavioral precautions. To the best of our knowledge, our work was the first to use a hypothesis-driven and pathway-based approach to study the association between genetic variations in the ROS pathway and risk of melanoma.

## 2. Results

### 2.1. Study Participants

Gender and age distributions of melanoma patients and healthy controls are listed in Table 1. In total, 177 retrieved cases and 172 recruited controls were approximately matched for age groups and gender. Overall, there are higher percentages of female patients aged 19–39 (55.4%) and 40–59 (26.5%), while, at age 60 and older, there is a higher percentage of male patients (47.9%). This may reflect the actual sex ratios of melanoma incidence at different age groups [23]. Of particular note, cases were retrieved from the international Genes, Environment, and Melanoma (GEM) study, which may not be strictly generalizable to a broader melanoma patient population. 

**Figure 2:**
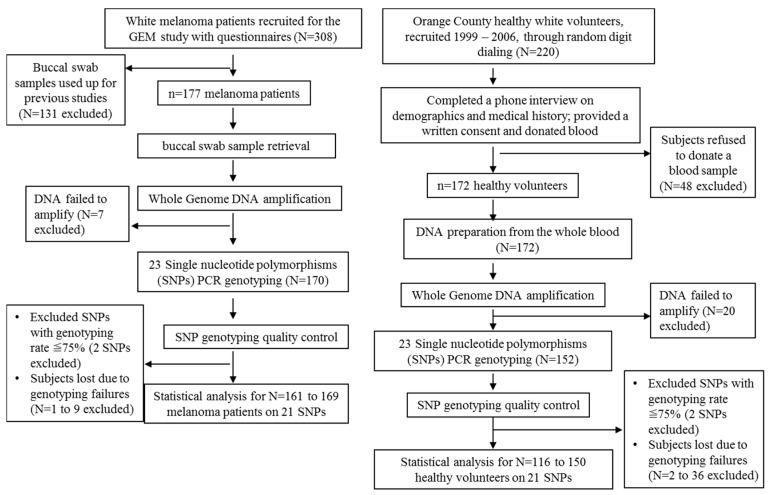
The inclusion and exclusion criteria of the participants in this study.

SNP candidates and their currently known disease associations are listed in Table 2. Whole genome DNA amplification was successfully carried out in 322 study participants including 170 (96%) melanoma patients and 152 (88.4%) healthy controls (Figure 2). However, for each SNP, there were different number of failed genotyping samples due to poor PCR reaction, and the overall successful genotyping rates were between 66.4% and 98.7% in the controls, and between 78.8% and 99.4% in the cases. SNPs with genotyping rate less than 75% on either arm (case or control group) of the participants were thus excluded from further analyses (SNPs rs13306296 and rs585197 were excluded, Table 3). Ultimately, 161–169 melanoma patients, and 116–150 healthy controls remained to be further analyzed (Figure 2).

### 2.2. SNP Associations

Chi-square or Fisher’s exact test of independence was performed to identify SNP frequency differences between melanoma patients and healthy controls under genotypic, allelic, recessive, and dominant SNP models (Table 3). An exact test of genotype counts on Hardy–Weinberg equilibrium (HWE) was conducted to identify and exclude SNPs not in genotype balance in our study sample. Under the genotypic model, five SNPs exhibited statistically significant (*p* < 0.05) frequency differences between cases and controls: rs10951982 (*RAC1*), rs8031 (*SOD2*), rs2536512 (*SOD3*), rs4673 (*CYBA*), and rs1049255 (*CYBA*) (Table 3). The allelic model only determined three of them as being significant: rs10951982 (*RAC1*), rs8031 (*SOD2*), and rs2536512 (*SOD3*). These three alleles exhibited significance in the recessive model as well. In the dominant model, rs10951982 (*RAC1*), rs4673 (*CYBA*), and rs1049255 (*CYBA*) showed significance. The rs1001179 (*CAT*) showed a significant difference between cases and controls in the dominant and recessive models but the significance disappeared in the other two models.

### 2.3. Bivariate Logistic Regression Analyses

The top five SNPs identified from the genotypic model without HWE violations were fitted into bivariate logistic regressions with additive, recessive, and dominant allele models, respectively. The odds ratios of melanoma risk were calculated using the homozygous major allele genotype as the reference (Table 4). Odds ratios derived from the regression models were compared to a corrected significance level at 0.00238 (0.05/21) to justify for multiple comparisons among the remaining 21 SNP candidates. Odds ratios with *p*-values < 0.00238 were considered having statistical significance in the results. 

In the additive allele model, carrying one copy of minor allele A in rs10951982 (*RAC1*) was significantly associated with a higher risk of melanoma (OR 8.98, 95% CI: 5.08, 16.44, *p* < 0.001), as compared to those who carried homozygous minor alleles AA (OR 8.23, 95% CI: 2.73, 28.39, *p* < 0.001). Dominant allele model further showed that combined minor allele copies (GA+AA) as compared to homozygous major alleles GG exhibited the highest risk of melanoma (OR 8.91, 95% CI: 5.09, 16.19, *p* < 0.001). A similar result was observed in rs4673 (*CYBA*), with one copy of the minor allele A exhibiting a higher risk of melanoma (OR 1.96, 95% CI: 1.23, 3.15, *p* = 0.005), and further confirmed in a dominant allele model (OR 1.84, 95% CI: 1.16, 2.92, *p* = 0.010). However, the p-values did not reach the corrected significance level of 0.00238.

The unadjusted odds of melanoma increased with homozygous minor allele T in rs1049255 (*CYBA*). TT exhibited an OR of 2.44 (95% CI: 1.27, 4.79, *p* = 0.008) in the additive model and an OR of 1.97 (95% CI: 1.10, 3.61, *p* = 0.022) in the recessive model. In both scenarios, *p*-values were greater than 0.00238, thus were non-significant because of the stringent Bonferroni correction for multiple comparison. 

In contrast, homozygous minor allele genotypes at both rs8031 (*SOD2*) and rs2536512 (*SOD3*) exhibited significant association with a reduced risk of melanoma in the additive allele model, with 84% reduction in odds of melanoma (OR 0.16, 95% CI: 0.06, 0.39, *p* < 0.001) for rs8031 (*SOD2*), and 92% reduction in odds of melanoma (OR 0.08, 95% CI: 0.01, 0.31, *p* = 0.001) for rs2536512 (*SOD3*). Similar results were also observed in the recessive model, where an 85% reduction in the odds of melanoma (OR 0.15, 95% CI: 0.06, 0.33, *p* < 0.001) was observed for rs8031 (*SOD2*) with TT minor alleles, and a 91% reduction (OR 0.09, 95% CI: 0.01, 0.33, *p* = 0.002 with marginal significance) for rs2536512 (*SOD3*) with AA minor alleles.

### 2.4. Multivariate Logistic Regression Analyses

We continued to fit these top five SNPs into multivariate logistic regression under the three SNP models, controlling for major melanoma risk factors including gender, age at diagnosis, family history of melanoma, and lifetime ever-sunburned (Table 5). After adjusting for these risk factors, rs1049255 (*CYBA*), rs4673 (*CYBA*), rs8031 (*SOD2*), and rs2536512 (*SOD3*) were no longer associated with melanoma risk in all three models (*p* > 0.00238).

Consistent with what we have found in Table 4, the most significant genotype was heterozygous GA genotype in rs10951982 (*RAC1*), which exhibited an OR of 6.15 (95% CI: 2.98, 13.44, *p* < 0.001) after controlling for other risk factors. This minor allele also showed a significant association with melanoma risk in the dominant model (OR 5.79, 95% CI: 2.84, 12.51, *p* < 0.001). Similar results were also found for rs4673 (*CYBA*) but with only marginal significance. Heterozygous GA genotype was associated with an increased risk of melanoma (OR 2.17, 95% CI: 1.17, 4.07, *p* = 0.015), which was further confirmed in the dominant allele model (OR 1.88, 95% CI: 1.03, 3.47, *p* = 0.042), although the *p*-values did not reach the corrected significance level of 0.00238. 

The homozygous minor allele TT genotype in rs8031 (*SOD2*) was found associated with a decreased risk of melanoma, with an OR of 0.32 (95% CI: 0.09, 0.94, *p* = 0.047) in the additive model, and an OR of 0.26 (95% CI: 0.08, 0.70, *p* = 0.011) in the recessive allele model, which indicated that homozygous minor alleles TT reduced the odds of melanoma by 74%, but neither of these results reached the universal significance level of 0.00238.

## 3. Discussion

After removal of SNP markers with high error rates during the assessment of genotyping quality, 21 SNP candidates remained to be eligible for the genetic association analysis. Eight SNPs showed significant association with melanoma but three of them were not in Hardy–Weinberg equilibrium, which may suggest that there are multiple alleles in the same locus, and we missed genotyping of other alleles. Therefore, only five SNP candidates showed genotypic significance and were further analyzed in regression models, including rs10951982 (*RAC1*), rs1049255 (*CYBA*), rs4673 (*CYBA*), rs8031 (*SOD2*), and rs2536512 (*SOD3*). We corrected the universal *p*-value to be compared with at 0.00238 (0.05/21, 21 SNPs being tested) to justify the multiple comparison issue in genetic association studies, using a Bonferroni approach [48,49]. The rs10951982 (*RAC1*) and rs4673 (*CYBA*) exhibited the highest increased risk of melanoma when presenting one copy of the minor allele in the unadjusted regression model, but rs4673 did not reach the universal significance level at 0.00238 in the multivariate regression model with adjustments for melanoma risk factors including age, sex, family history of melanoma, and lifetime ever-sunburned. Of particular note, a homozygous minor allele TT genotype in rs8031 (*SOD2*) was found to be associated with reduced risk of melanoma in the bivariate regression, however significance was lost in the multivariate regression analyses. 

SOD2 is known to be a major superoxide detoxifying enzyme of cells, and therefore an altered function or expression of this enzyme may lead to unbalanced redox homeostasis and thus potentially increase or decrease the risk of melanoma [40]. Since SOD2 converts superoxide to hydrogen peroxide (Figure 1), which belongs to a type of ROS, the function of SOD2 is thus double-edged. Our multivariate analysis indicated that homozygous TT allele in rs8031 reduced the risk of melanoma, but little is currently known about the molecular function of this variant. We suggest a lab-based functional molecular biology study to unravel the discrepancy between zygote expression and enzymatic activity in this particular SNP. 

SNPs rs1049255 and rs4673 in *CYBA* showed genotypic frequency differences between cases and controls in the unadjusted model (Table 4), with more patients carrying higher copies of minor alleles in rs1049255. Variant rs4673 changes the amino acid at position 72 from a tyrosine to a histidine (Y72H) of the CYBA (p22phox) protein, which is frequently referred to a C242T variant in the literature [50]. The T allele exhibited decreased dimerization with NOX and therefore may potentially reduce NOX activity and cellular ROS level [32]. In fact, the CT and TT genotype showed lower NADPH oxidase activity in hypertensive patients as compared with CC genotype [51]. However, opposite observation was also reported, where the CT genotype and T allele are associated with higher risk of coronary artery disease [52]. In our study, the CT and TT (GA and AA) showed higher risk for melanoma as compared to CC (GG) allele (the dominant model in Table 4 and Table 5). This observation needs further validation. Variant rs1049255 is located in the 3′ untranslated region (3′ UTR) of the *CYBA* gene. Although the molecular function of this SNP is unknown, current understanding of 3′ UTR is an important miRNA binding site, and SNPs located in this region might have the potential to regulate mRNA stability and translation efficiency [53,54].

RAC1-GTPase is an NOX1 activator which promotes binding of NOX1 with its subunits and forms the complete enzyme complex [55,56,57]. NOX1 was one of the first cellular molecules found to be directly regulated by RAC1 in the phagocytic process [58,59,60]. However, SNP rs10951982 in *RAC1* alone has not been reported in any ROS-related activities thus far. Information on the function of this locus and its association with any malignancy is limited in the current literature. Nevertheless, this variant has been reported to be associated with over-reactive immune diseases and an increased risk of hypertension [20,35,36,61]. Considering that *CYBA* variants have been widely studied in cardiovascular diseases, including coronary heart disease [34] and hypertension [33], which are tightly associated with increased levels of ROS, *RAC1* rs10951982 may also play a part in inducing oxidative stress. Since rs10951982 is the most significant variant in our current study, and in lieu of its function in immune diseases as well as a potential role in NOX1-induced oxidative stress, our discovery might not only suggest an inflammatory microenvironment created by RAC1 that is in favor of melanoma progression [62], but also indicate an elevation of ROS level via RAC1 in melanoma etiology. In addition, RAC1 is also a crucial kinase in the NRAS and PI3K pathway [63], both of which are key melanoma oncogenic pathways. Therefore, it is possible that RAC1 plays a non-ROS role and impacts these other oncogenic pathways. 

Overall, of the three significant SNPs after adjustment against age, sex, family history and life time sun burn history, the minor allele of *RAC1* rs10951982 (the A allele) showed a consistent role with an increase ROS and thus increased melanoma risk. The minor allele of rs4673 (the A allele) was reported controversial role in ROS association [51,64], it may exhibit certain cell-specific effects. In our study, the minor allele showed higher risk for melanoma in a dominant model. The minor allele of rs8031 (the T allele) exhibited a protective role against melanoma risk in a recessive model. It is unclear how this allele modifies ROS levels. Based on our results, the T allele can be associated with either increased or decreased SOD activities as SOD2 is double-edged and can play dual roles in ROS metabolism. 

Of particular note, in our regression models, we applied the most common ways of disease transmission, namely additive, recessive, and dominant modes, in our analyses. This was because we did not want to make any assumptions of the disease transmission modes. According to Sham and Purcell [49], a test that assumed additive effects would have greater power than a test that also allowed dominance, if the true effects at the locus were indeed additive and did not show dominance. Conversely, if the underlying causal variant was recessive, then power would be lost by carrying out an analysis that assumed additively. If there was uncertainty regarding the true pattern of effects at a locus, then it might be appropriate to use several statistical tests to ensure adequate statistical power for all possible scenarios. We therefore included results from these additional models that may provide more information and maintain statistical power as well. Although the covariates were not presented as part of the results in our tables, family history of melanoma and lifetime ever-sunburned controlled in the multivariate models consistently showed statistical significance, whereas sex and age did not. Family history of melanoma [23], along with fair skin, light hair and eye color are known melanoma genetic risk factors, whereas the levels of sun exposure including sunburns and moles or freckles are important environmental risk factors for melanoma [65]. The statistical significance of the covariates might indicate a mediating role in our primary study interest, from the susceptible familial genetic makeup of these participants, as well as the behavior or attitude towards sun exposure that resulted in getting sunburns or freckles. 

Our study had a few limitations. First, the small sample size does not always provide sufficient power [66]. Second, by the experimental design, we could only genotype two alleles. Therefore, loci with multiple alleles may not show HWE and must be excluded for analysis. Third, our study participants included only those white individuals from the southern California area, and therefore a loss of generalizability to the broader white population might be expected. Last, a common limitation of case-control studies is that the results provide only an association with risk, but they are not necessarily connected to causality. Replicating findings from another dataset is a common strategy to validate the results identified in our current study. However, even with the most stringent statistical design, SNP findings are usually hard to replicate [48,49]. Multiple reasons are considered, such as there are still unknown and uncontrolled confounders, multiple comparisons only lead to chance findings, the gene and environment interaction is not easy to account for, and the target allele is in linkage disequilibrium with the identified allele but the chance finding failed to locate the target allele and thus make replication difficult to achieve. Nevertheless, we will still validate our findings in a separate dataset in our next study, as our ultimate goal is to develop useful markers in prevention. 

To conclude, our initial analyses revealed an increased risk of melanoma associated with rs10951982 (*RAC1*), and a decreased risk associated with rs8031 (*SOD2*). Multivariate analyses further confirmed the association of an increased risk of melanoma with rs10951982 (*RAC1*). Our results highlighted the importance of RAC1 enzyme and cellular oxidation-metabolizing efficiency controlled by SOD2 in association with ROS-mediated risk of melanoma. We suggest that these results shall be further validated with the goal of designing novel screening targets to identify highly UV-susceptible individuals, particularly in the *RAC1* and *SOD2* genes, in order to take the melanoma primary prevention strategy to a precision level.

## 4. Materials and Methods

### 4.1. Ethics Statement

We obtained approval from the Institutional Review Board of the University of California Irvine Office of Research (protocol number 2011-8238, approved 27 June 2011).

### 4.2. Study Population

Our study subjects were adopted from a previously designed case-control study (the international Genes, Environment, and Melanoma study, the GEM study), although we made considerable modifications. The original GEM case-control study compared white multiple melanomas patients to primary melanoma patients [67]. In total, 177 patients were recruited between 1998 and 2003 in the southern California area as part of the GEM study, and consent forms were obtained accordingly [67]. In our study, we used both of these patients as our cases and we recruited additional healthy participants as controls. Healthy white volunteers from Orange County were recruited through random-digit-dialing by trained interviewers during 1999 to 2006. 

Demographic information regarding age, sex, family history of melanoma, and lifetime sun exposure were recorded via in-person questionnaires and phone interviews, with written consents from the patients and their physicians [67,68,69,70,71,72,73]. Random-digit-dialing healthy respondents completed eligibility screening questions over the phone, including being Orange County residents and having no personal history of melanoma or any other types of cancer. Eligible respondents were asked for their verbal informed consents for a 20 min standardized phone interview [67], in which they were asked questions about basic demographics, personal medical history, and family cancer history. In total, 172 participants further agreed to donate a blood sample. A phlebotomist obtained written consents from these participants while performing the blood draw [67]. Participation rate after phone eligibility screening was approximately 78%. Population-based controls were frequency-matched to cases with respect to sex and age (Table 1).

### 4.3. DNA Extraction

Buccal cells from melanoma patients and whole blood cells from healthy participants were re-suspended in a phosphate-buffered saline system. Ten microliters of the cell suspension were used directly as a template for whole genome amplification (WGA). The WGA procedure was conducted following the manufacturer’s instruction from Sigma. In brief, a cell suspension (10 μL each) was heated to 95 °C for 5 min in a PCR machine in a strip of PCR tubes and cooled down on ice. One microliter of 10× Fragmentation Buffer was added to each tube. Tubes were then heated again in a PCR machine at 95 °C for exactly 4 min. Samples were cooled down on ice immediately and then centrifuged briefly to consolidate the contents. Out of 70 μL of the amplified sample, 6 μL was mixed with 1 μL of 6× loading buffer and directly used to load on an agarose DNA gel containing ethidium bromide. DNA was visualized under a UV lamp and water was used as a non-DNA negative control to compare with the presence of the visualized DNA product. Participants with little to no whole genome amplified DNA product were excluded from SNP genotyping (7 patients and 20 healthy controls were excluded, Figure 2).

### 4.4. SNP Candidates

Functional SNPs were selected from a publicly available SNP database (dbSNP, NCBI) that have been found correlated with other diseases, based on the three criteria listed in the introduction (Table 2). In brief, 6 SNPs in the coding region of *NOX1* appeared in dbSNP. We were interested in D360N (rs34688635) and R315H (rs2071756) variants for the following reasons: (1) D360 is shared in *NOX1*, *-2*, *-3*, and *-4* [25], and conserved in various species including fish, mouse, bird, amphibian, and man [9]; and (2) 315H allele was found associated with diabetic patients, suggesting that this is a functional allele and may be associated with other disease risks [24]. SNPs rs585197 and rs2164521 in *NOX4* have been linked to a protective effect on Hepatopulmonary Syndrome [27]; and rs11018628 has a possible effect on plasma homocysteine level [26]. −930A > G in *CYBA* promoter region (rs9932581) affects gene transcription activity and has been found to be associated with coronary heart disease due to ROS involvement in the pathogenesis of atherosclerosis [28]. Similarly, increased or decreased risks of hypertension [33] and coronary heart disease [34], respectively, have been found in *CYBA* alleles rs4673, rs13306296, and rs1049255. *CYBA* rs3180279 has been related to non-Hodgkin lymphoma prognosis [13]. Three SNPs, rs10951982, rs4720672, and rs836478 in *RAC1*, have been associated with risks in hypertension, inflammatory bowel disease, and end-stage renal disease [20,35,36,61]. Although these loci in *RAC1* have not yet been discussed in ROS-related malignancies, *RAC1* is a well-known melanoma oncogene with constantly activated mutations in some melanoma tumors [74,75]. 

SNPs in the three subtypes of *SOD* and *CAT* genes have been widely studied with various disease associations. For instance, rs7277748 and rs4998557 variants in *SOD1* (Cu-ZnSOD) were found to cause amyotrophic lateral sclerosis. Ile58Thr (rs1141718) in *SOD2* (MnSOD) severally impaired SOD2 enzymatic activity [40], while a variant of rs8031 increased oxidative stress [44]. V16A variant rs4880 in *SOD2* impaired mitochondrial importing and was found to be a risk factor for prostate cancer [12], whereas rs2758330 showed a protective effect on prostate cancer [45]. Variants rs2536512 and rs699473 in *SOD3* were associated with brain diseases, including cerebral infarction [14] and brain tumor [15]. The rs1001179 in *CAT* was also correlated to brain malignancy [15]. Additionally, −262C > T (rs1049982) variant in *CAT* showed a decreased interaction with HIF1α upon oxidative stress stimulation [46,47] (Table 2).

### 4.5. SNP Genotyping

SNP genotyping polymerase chain reaction (PCR) assay kit was purchased from Life Technologies™ (Carlsbad, CA, USA). Allele-specific primers and probe sets for each SNP were also purchased from Life Technologies™, either custom-designed or from the library. DNA sample per participant was genotyped for every SNP in duplicates to ensure accuracy. About 97% of the SNPs were replicable. By definition, if one allele was amplified during PCR reaction, the call for that SNP assay was homozygous alleles (inherited the same alleles from both parents); if both alleles were amplified, the call for that SNP assay was heterozygous alleles (inherited different alleles from the parents). However, if no significant PCR amplification for either allele was observed, then the SNP assay was defined as N/A (genotyping failure) due to no reaction to the designed allele primers and probe. SNPs with genotyping rate < 75% were excluded from statistical analysis (SNPs rs13306296 and rs585197 were excluded from further analysis, Table 3). SNPs with inconsistent duplicated results were validated manually by reading the raw real-time PCR amplification plots, or through additional genotyping reactions.

### 4.6. SNP Quality Control

The raw PCR amplification data was analyzed by QuantStudio™ (Thermo Fisher Scientific Inc., Huntington Beach, CA, USA) Real-Time PCR software (v1.2). Those duplicated samples presenting identical calls were automatically determined by the software. However, if the calls were made differently between duplicates, or, in some rare cases, if the calls were “undetermined” by the software, then the individual PCR amplification plots were read manually and subjectively. Any amplification curve appearing after 20 cycles of PCR, and being at least two-fold elevated from the threshold was determined as presenting a positive PCR amplification curve. Genotyping failure was assigned as N/A if no clear PCR amplification curve was observed.

### 4.7. Statistics

Allele frequency was determined by making counts of the participants based on different SNP conditions: genotypic, allelic, recessive, and dominant models. Chi-square or Fisher’s exact test of independence was performed to examine the associations between SNP conditions and melanoma case-control status. Two-sided statistical significance level by default was set to be 0.05 (5%), and, to justify for multiple comparison among the SNP candidates, universal significance level was further adjusted to 0.05 divided by the number of final SNP candidates being tested, which was 0.05/21 = 0.00238, applying the most stringent Bonferroni approach [48,49]. Participant numbers varied among SNPs due to different genotyping rates, and only complete data was used for statistical analysis (participants with N/A data were excluded per SNP analysis). Bivariate simple logistic regression models showing the unadjusted associations between the binary response variable (melanoma cases vs. controls) and primary study variables of interest (SNPs) were conducted separately based on additive, recessive, and dominant allele models. Dummy variables of the SNPs in the three allele models were created by default, making genotype with homozygous major alleles as the reference to compare with. Odds ratios and 95% confidence intervals were calculated accordingly in RStudio (v0.99.893). Adjusted associations between SNPs and melanoma status were analyzed by fitting multivariate logistic regression models with the three allele models separately, controlling for known melanoma risk factors, including gender [76], age at diagnosis [77], family history of melanoma [23], and ever sunburned [78]. Genotypic Hardy–Weinberg equilibrium exact test, which examines the expected frequencies of genotypes if mating is non-assortative and there are no mutations from one allele to another, was carried out by using R package HardyWeinberg. In brief, a two-sided test was performed on genotype counts, whether an excess or a dearth of heterozygotes counts as evidence (*p* < 0.05) against Hardy–Weinberg equilibrium.

## Figures and Tables

**Table 1:** Characteristics of the study participants.

Study Participant	Gender		
	Male	Female	Total
	*n* (%) ^1^	*n* (%)	*n* (%)
Patients (*n* = 177)			
Age (years)			
19–39	5 (5.32%)	15 (18.1%)	20 (11.3%)
40–59	44 (46.8%)	46 (55.4%)	90 (50.8%)
≥60	45 (47.9%)	22 (26.5%)	67 (37.9%)
Controls (*n* = 172)			
Age (years)			
19–39	7 (7.1%)	15 (20.3%)	22 (12.8%)
40–59	45 (45.9%)	41 (55.4%)	86 (50.0%)
≥60	46 (46.9%)	18 (24.3%)	64 (37.2%)

^1^ Percentage may not add up to 100% due to rounding.

**Table 2:** Twenty-three SNP candidates.

Gene	SNP	location	dbSNP ID	Disease Association	Reference
*NOX1*	944G>A	R315H	rs2071756	Diabetes	[24]
	1284G>A	D360N	rs34688635	Severe pancolitis	[25]
*NOX4*	T>C	Intron	rs11018628	Increased plasma homocysteine level (risk in cardiovascular diseases)	[26]
	−114 C>T	5’UTR	rs585197	Decreased risk of hepatic-pulmonary syndrome	[27]
	C>T	Intron	rs2164521	Decreased risk of hepatic-pulmonary syndrome	[27]
*CYBA^p22phox^*	−930A>G	Promoter	rs9932581	Modulates CYBA promoter activity	[28,29]
	242C>T	Y72H	rs4673	Decreased NOX activity; protective role in coronary heart disease	[30,31,32]
	−675A>T	Promoter	rs13306296	Related to hypertension	[33]
	C>G	Intron	rs3180279	Associated with non-Hodgkin lymphoma prognosis	[13]
	640A>G	3′UTR	rs1049255	Associated with coronary heart disease	[34]
*RAC1*	G>A	Intron	rs10951982	Risks in ulcerative colitis, hypertension, inflammatory bowel disease, end-stage renal disease	[20]
	T>C	Exon	rs4720672	Risks in inflammatory bowel disease, ulcerative colitis	[35,36]
	C>T	Intron	rs836478	Hypertension risk factor	[20]
*SOD1*	A>G	5’UTR	rs7277748	Familial amyotrophic lateral sclerosis	[37]
	7958G>A	Intron	rs4998557	Caused amyotrophic lateral sclerosis	[37,38,39]
*SOD2*	399T>C	Ile58Thr	rs1141718	Reduced enzyme activity	[40]
	T>C,A,G	V16A,D,G	rs4880	Mitochondrial importing, diabetes and prostate cancer	[12,41,42,43]
	T>A	Intron	rs8031	Oxidative stress	[44]
	C>A	Intron	rs2758330	Protective role in prostate cancer	[45]
*SOD3*	C>T	Promoter	rs699473	Brain tumor	[15]
	G>A	A377T	rs2536512	Cerebral infarction	[14]
*CAT*	−262C>T	5’UTR	rs1049982	Down-regulated transcription upon oxidative stimulation	[46,47]
	C>T	5’UTR	rs1001179	Brain tumor	[15]

**Table 3:** Descriptive statistics of the 23 SNP candidates.

SNP ^1^	Gene	Genotyping Rate ^2^		Minor Allele Frequency (MAF)		Association (*p*-Value) ^4^				HWE ^5^ (*p*-Value)	dbSNP MAF ^6^
		Cases (*n* = 170) ^3^	Controls (*n* = 152) ^3^	Cases	Controls	Genotypic	Allelic	Recessive	Dominant		
rs10951982	*RAC1*	96.5%	83.0%	47.3%	23.6%	<0.001	<0.001	0.333	<0.001	0.459	16.6%
rs11018628	*NOX4*	99.4%	94.1%	50.0%	33.9%	<0.001	<0.001	0.458	<0.001	<0.001	16.7%
rs8031	*SOD2*	95.9%	87.5%	38.3%	49.6%	<0.001	0.008	<0.001	0.576	0.605	36.7%
rs2536512	*SOD3*	97.1%	75.8%	27.6%	37.5%	<0.001	0.016	<0.001	0.168	0.431	40.1%
rs4720672	*RAC1*	96.5%	92.2%	23.8%	17.6%	0.009	0.076	0.582	0.014	0.043	12.5%
rs4673	*CYBA*	98.8%	93.5%	36.6%	30.6%	0.014	0.132	0.561	0.013	0.238	33.6%
rs3180279	*CYBA*	98.8%	94.7%	48.2%	48.6%	0.022	0.985	0.154	0.127	0.030	44.5%
rs1049255	*CYBA*	97.1%	90.3%	48.2%	37.1%	0.027	0.007	0.034	0.041	0.719	46.9%
rs1001179	*CAT*	96.5%	95.4%	16.8%	24.1%	0.062	0.030	0.610	0.025	1.000	12.6%
rs4880	*SOD2*	98.2%	93.5%	53.3%	55.2%	0.074	0.685	0.133	0.402	0.175	41.1%
rs9932581	*CYBA*	99.4%	90.9%	45.9%	42.5%	0.357	0.450	1.000	0.212	0.168	41.7%
rs699473	*SOD3*	97.1%	90.9%	63.0%	59.3%	0.461	0.388	0.745	0.280	0.861	44.1%
rs7277748	*SOD1*	95.9%	85.6%	4.0%	2.7%	0.486	0.518	N/A	0.511	1.000	3.9%
rs2164521	*NOX4*	98.2%	86.8%	9.3%	10.6%	0.646	0.688	0.442	0.792	1.000	26.2%
rs836478	*RAC1*	97.1%	94.8%	51.8%	50.0%	0.740	0.710	0.551	1.000	1.000	30.9%
rs4998557	*SOD1*	98.8%	94.1%	10.4%	11.5%	0.749	0.774	1.000	0.675	0.695	32.9%
rs1049982	*CAT*	94.7%	88.8%	35.7%	36.3%	0.962	0.951	1.000	0.904	0.710	47.1%
rs34688635	*NOX1*	97.6%	90.2%	1.5%	1.1%	1.000	0.734	1.000	1.000	1.000	0.5%
rs1141718	*SOD2*	96.5%	98.7%	49.4%	49.0%	1.000	0.988	1.000	1.000	<0.001	N/A
rs2758330	*SOD2*	96.5%	96.1%	20.7%	20.6%	1.000	1.000	1.000	1.000	0.010	26.5%
rs2071756	*NOX1*	98.2%	92.2%	0%	0%	N/A	1.000	N/A	N/A	1.000	0.1%
rs585197	*NOX4*	73.5%	70.4%	Excluded from further analysis due to low genotyping rate (≤75.0%)							
rs13306296	*CYBA*	78.8%	66.4%	Excluded from further analysis due to low genotyping rate (≤75.0%)							

^1^ Ordered according to smallest to largest genotypic *p*-values; ^2^ Percentage of participants with SNP genotyping success; ^3^ Participant number = *n* * %; ^4^ Chi-square or Fisher’s exact test of independence between SNP models and melanoma status (case and control); ^5^ Exact test for Hardy–Weinberg equilibrium (HWE) on the controls, *p* < 0.05 counts as evidence against HWE. HWE is a test of genotype balance in a given population; ^6^ Reference minor allele frequencies documented in dbSNP database. N/A: not available.

**Table 4:** Crude associations between the top five SNPs and melanoma risk.

SNP/Model	Allele	Cases (*n* = 170)	Controls (*n* = 152)	OR (95% CI)	*p*-Value ^2^
		*n* (%) ^1^	*n* (%)		
**rs10951982** (*RAC1*)					
Additive	GG	21 (12.4%)	72 (47.4%)	Reference	--
	GA	131 (77.1%)	50 (32.9%)	8.98 (5.08, 16.44)	<0.001
	AA	12 (7.1%)	5 (3.3%)	8.23 (2.73, 28.39)	<0.001
Recessive	GG+GA	152 (89.4%)	122 (80.3%)	Reference	--
	AA	12 (7.1%)	5 (3.3%)	1.93 (0.69, 6.19)	0.230
Dominant	GG	21 (12.4%)	72 (47.4%)	Reference	--
	GA+AA	143 (84.1%)	55 (36.2%)	8.91 (5.09, 16.19)	<0.001
**rs1049255** (*CYBA*)					
Additive	CC	47 (27.6%)	56 (36.8%)	Reference	--
	CT	77 (45.3%)	63 (41.4%)	1.46 (0.88, 2.44)	0.149
	TT	41 (24.1%)	20 (13.2%)	2.44 (1.27, 4.79)	0.008
Recessive	CC+CT	124 (72.9%)	119 (78.3%)	Reference	--
	TT	41 (24.1%)	20 (13.2%)	1.97 (1.10, 3.61)	0.022
Dominant	CC	47 (27.6%)	56 (36.8%)	Reference	--
	CT+TT	118 (69.4%)	83 (54.6%)	1.69 (1.05, 2.74)	0.031
**rs4673** (*CYBA*)					
Additive	GG	53 (31.2%)	66 (43.4%)	Reference	--
	GA	107 (62.9%)	68 (44.7%)	1.96 (1.23, 3.15)	0.005
	AA	8 (4.7%)	10 (6.6%)	1.00 (0.36, 2.70)	0.994
Recessive	GG+GA	160 (94.1%)	134 (88.2%)	Reference	--
	AA	8 (4.7%)	10 (6.6%)	0.67 (0.25, 1.75)	0.412
Dominant	GG	53 (31.2%)	66 (43.4%)	Reference	--
	GA+AA	115 (67.6%)	78 (51.3%)	1.84 (1.16, 2.92)	0.010
**rs8031** (*SOD2*)					
Additive	AA	45 (26.5%)	32 (21.1%)	Reference	--
	AT	111 (65.3%)	70 (46.1%)	1.13 (0.65, 1.94)	0.665
	TT	7 (4.1%)	31 (20.4%)	0.16 (0.06, 0.39)	<0.001
Recessive	AA+AT	156 (91.8%)	102 (67.1%)	Reference	--
	TT	7 (4.1%)	31 (20.4%)	0.15 (0.06, 0.33)	<0.001
Dominant	AA	45 (26.5%)	32 (21.1%)	Reference	--
	AT+TT	118 (69.4%)	101 (66.4%)	0.83 (0.49, 1.40)	0.489
**rs2536512** (*SOD3*)					
Additive	GG	76 (44.7%)	43 (28.3%)	Reference	--
	GA	87 (51.2%)	59 (38.8%)	0.83 (0.51, 1.37)	0.477
	AA	2 (1.2%)	14 (9.2%)	0.08 (0.01, 0.31)	0.001
Recessive	GG+GA	163 (95.9%)	102 (67.1%)	Reference	--
	AA	2 (1.2%)	14 (9.2%)	0.09 (0.01, 0.33)	0.002
Dominant	GG	76 (44.7%)	43 (28.3%)	Reference	--
	GA+AA	89 (52.4%)	73 (48.0%)	0.69 (0.42, 1.12)	0.134

^1^ Participants lost due to genotyping failure; ^2^ *p*-value of the coefficient from the regression model. *p*-value was compared to a Bonferroni corrected significance level at 0.05/21 = 0.00238 to determine statistical significance. --: no *p*-value in the reference group.

**Table 5:** Adjusted ^1^ associations between the top five SNPs and melanoma risk.

SNP/Model	Allele	Cases (*n* = 170)	Controls (*n* = 152)	OR (95% CI)	*p*-Value ^3^
		*n* (%) ^2^	*n* (%)		
**rs10951982** (*RAC1*)					
Additive	GG	21 (12.4%)	72 (47.4%)	Reference	--
	GA	131 (77.1%)	50 (32.9%)	6.15 (2.98, 13.41)	<0.001
	AA	12 (7.1%)	5 (3.3%)	2.88 (0.68, 12.56)	0.149
Recessive	GG+GA	152 (89.4%)	122 (80.3%)	Reference	--
	AA	12 (7.1%)	5 (3.3%)	0.79 (0.21, 3.03)	0.719
Dominant	GG	21 (12.4%)	72 (47.4%)	Reference	--
	GA+AA	143 (84.1%)	55 (36.2%)	5.79 (2.84, 12.51)	<0.001
**rs1049255** (*CYBA*)					
Additive	CC	47 (27.6%)	56 (36.8%)	Reference	--
	CT	77 (45.3%)	63 (41.4%)	1.20 (0.63, 2.30)	0.574
	TT	41 (24.1%)	20 (13.2%)	1.42 (0.61, 3.38)	0.420
Recessive	CC+CT	124 (72.9%)	119 (78.3%)	Reference	--
	TT	41 (24.1%)	20 (13.2%)	1.28 (0.59, 2.83)	0.531
Dominant	CC	47 (27.6%)	56 (36.8%)	Reference	--
	CT+TT	118 (69.4%)	83 (54.6%)	1.26 (0.69, 2.31)	0.456
**rs4673** (*CYBA*)					
Additive	GG	53 (31.2%)	66 (43.4%)	Reference	--
	GA	107 (62.9%)	68 (44.7%)	2.17 (1.17, 4.07)	0.015
	AA	8 (4.7%)	10 (6.6%)	0.50 (0.11, 1.82)	0.315
Recessive	GG+GA	160 (94.1%)	134 (88.2%)	Reference	--
	AA	8 (4.7%)	10 (6.6%)	0.31 (0.07, 1.07)	0.080
Dominant	GG	53 (31.2%)	66 (43.4%)	Reference	--
	GA+AA	115 (67.6%)	78 (51.3%)	1.88 (1.03, 3.47)	0.042
**rs8031** (*SOD2*)					
Additive	AA	45 (26.5%)	32 (21.1%)	Reference	--
	AT	111 (65.3%)	70 (46.1%)	1.33 (0.66, 2.65)	0.421
	TT	7 (4.1%)	31 (20.4%)	0.32 (0.09, 0.94)	0.047
Recessive	AA+AT	156 (91.8%)	102 (67.1%)	Reference	--
	TT	7 (4.1%)	31 (20.4%)	0.26 (0.08, 0.70)	0.011
Dominant	AA	45 (26.5%)	32 (21.1%)	Reference	--
	AT+TT	118 (69.4%)	101 (66.4%)	1.06 (0.54, 2.08)	0.864
**rs2536512** (*SOD3*)					
Additive	GG	76 (44.7%)	43 (28.3%)	Reference	--
	GA	87 (51.2%)	59 (38.8%)	0.68 (0.35, 1.28)	0.232
	AA	2 (1.2%)	14 (9.2%)	0.26 (0.03, 1.50)	0.144
Recessive	GG+GA	163 (95.9%)	102 (67.1%)	Reference	--
	AA	2 (1.2%)	14 (9.2%)	0.33 (0.04, 1.83)	0.218
Dominant	GG	76 (44.7%)	43 (28.3%)	Reference	--
	GA+AA	89 (52.4%)	73 (48.0%)	0.65 (0.34, 1.21)	0.175

^1^ Adjusted for gender, age at diagnosis/interview, family history of melanoma, and ever sunburned; ^2^ Participants lost due to genotyping failure; ^3^ *p*-value of the coefficient from the regression model. *p*-value was compared to a Bonferroni corrected significance level at 0.05/21 = 0.00238 to determine statistical significance. --: no *p*-value in the reference group.

## Abbreviations

UV	Ultraviolet
ROS	Reactive oxygen species
SNP	Single nucleotide polymorphism
NOX	NADPH oxidase
SOD	Superoxide dismutase
CAT	Catalase
OR	Odds ratio
HWE	Hardy–Weinberg equilibrium
